# Cataract surgery and age-related cognitive decline: A 13-year follow-up of the English Longitudinal Study of Ageing

**DOI:** 10.1371/journal.pone.0204833

**Published:** 2018-10-11

**Authors:** Asri Maharani, Piers Dawes, James Nazroo, Gindo Tampubolon, Neil Pendleton, on behalf of the SENSE-Cog WP1 group

**Affiliations:** 1 Division of Neuroscience and Experimental Psychology, University of Manchester, Manchester, United Kingdom; 2 Division of Human Communication, Development & Hearing, University of Manchester, Manchester, United Kingdom; 3 Cathie Marsh Institute for Social Research, University of Manchester, Manchester, United Kingdom; Cardiff University, UNITED KINGDOM

## Abstract

**Background:**

Visual impairment has been associated with lower cognitive ability among older adults, yet little is known about whether improving visual function with cataract surgery would be associated with slower cognitive decline. This study aimed to assess whether trajectories of cognitive decline differed before and after cataract surgery and compare those trajectories between older adults with cataract surgery and without cataract.

**Methods and findings:**

Data were drawn from the English Longitudinal Study of Ageing (ELSA) Wave 1 (2002/03) until Wave 7 (2014/15). The study population consisted of 2,068 individuals who underwent cataract surgery between Wave 2 and Wave 6 as the treatment group and 3,636 individuals with no cataract as the control group. We included only respondents who took part in a minimum three waves. Propensity score matching method was used to match the individuals in the treatment group with those in the control group. After we put an “artificial” intervention point for the individuals in the control group at the point that the matched person has cataract surgery, spline method was used to identify differences in cognitive trajectories pre- and post-cataract surgery. In the treatment group, we found that cataract surgery was positively associated with episodic memory scores after controlling for the potential covariates (β = 4.23, *p*<0.001). Episodic memory scores declined with older age, but the decline in episodic memory scores was slower after cataract surgery (β = -0.05, *p*<0.001) than before cataract surgery (β = -0.1, *p*<0.001). Although the episodic memory among respondents in the control group before intervention (β = -0.08, *p*<0.001) declined slower than those in the intervention group (β = -0.1, *p*<0.001), the declines in episodic memory scores were similar in both groups after the intervention (control: β = -0.05, p<0.001; intervention: β = -0.05, p<0.001).

**Conclusions:**

Cataract surgery may have a positive impact on trajectories of cognitive decline in later life. Further research is required to identify the mechanism to explain the association between cataract surgery and cognitive ageing, and whether early intervention towards vision correction results in a reduction in dementia risk.

## Introduction

Visual impairment—a condition affecting one in three adults 50 years or older [1]–is one of the risk factors for cognitive decline and dementia. The presence of visual and hearing impairment is associated with lower cognitive function among older adults in Europe and the United States (US) [2]. A retrospective study examining 625 older adults in the US found that those with untreated poor vision had a ninefold risk of developing Alzheimer’s disease and a fivefold risk of developing cognitive impairment no dementia (CIND) [3].

In spite of significant effort invested in describing associations between visual impairment and cognitive performance/dementia among the older population, there is little evidence on the nature of the relationship between visual impairment and cognitive function or the impact of interventions for vision impairment on cognition. Three main hypotheses may explain the relationship between sensory (including vision and hearing) impairment and cognitive performance. The first is the common cause hypothesis, which suggests that the decline of sensory and cognitive functions share common age-related causes such as the degeneration of the central nervous system [4]. The second is the cascade hypothesis, according to which sensory impairment may have a “domino effect” on cognitive abilities [5], possibly through neurobiological mechanisms or mediated by lower self-efficacy, social isolation, or depression [4, 6]. An alternative third hypothesis is that sensory impaired individuals are disadvantaged in their cognitive test performance [7, 8]. Poor sensory function provides degraded perceptual input to the cognitive system, which leads to poorer performance on cognitive tests.

We modelled the impact of cataract surgery on cognitive decline in order to i) test the possibility that treating visual impairment could reduce the rate of cognitive decline, a major risk factor for dementia, and ii) test hypotheses that posit a causal impact of vision impairment on cognitive decline. Cataracts, or age-related opacification of the lens, are the commonest cause of reversible visual loss, affecting 16 million people worldwide [9]. Cataract intervention usually consists of surgery to replace the clouded crystalline lens with an intraocular lens (IOLs). Cataract surgery is the most common form of refractive surgery today; its benefits in terms of better vision and quality of life are well established [10].

Previous studies have reported the benefits of cataract surgery beyond visual outcomes, such as higher perceived health, lower anxiety symptoms, and better cognition. However, studies analysing the effect of cataract surgery on cognitive performance have been inconclusive: some studies have suggested that there are no effects [11, 12], whereas more recent studies have shown positive effects [13–15]. Most studies to date have incorporated short-term follow-ups, used community or volunteer samples rather than representative national samples, and included a limited set of risk factors beyond gender and age in their models. In the present study, we tested the hypothesis that cataract surgery would have a positive association with cognitive trajectories in community-dwelling older adults in two steps. We firstly modelled changes in cognitive trajectories pre- and post-cataract surgery among respondents who had cataract surgery between Wave 2 (2004/05) and Wave 6 (2012/13) of ELSA (treatment group) using measures of episodic memory scores taken at two-year intervals between 2002 and 2015. We then provided a control group (respondents who had no cataract disease until the last wave available) using propensity score matching method and modelled the similar cognitive trajectories for those with no cataract disease (control group).

## Materials and methods

This study forms part of the SENSE-Cog multi-phase research programme, funded by the European Union Horizon 2020 programme. SENSE-Cog aims to promote mental well-being in older adults with sensory and cognitive impairments (http://www.sense-cog.eu/). The first work package of this project aims to better understand the links between sensory, cognitive and mental ill-health in older Europeans.

### Study design and participants

We used seven waves of the English Longitudinal Study of Ageing (ELSA) spanning from 2002 to 2015 [16]. ELSA is a biannual, nationally representative, longitudinal study of men and women aged 50 and older in England providing information on demographics, socio-economics, health, and social participation. The ELSA ethical approval was obtained from the National Health Service Research Ethics Committees under the National Research and Ethics Service. The first wave of the study was conducted in 2002 with 11,391 respondents who had previously participated in the Health Survey for England 1998, 1999, and 2001. Refreshment samples were recruited in waves three, four, six, and seven. The conditional response rates in ELSA are 82% of respondents in wave 1 participated in wave 2, 73% in wave 3, 74% in wave 4, 80% in wave 5, 81% in wave 6 and 82% in wave 7. The details on conditional response rates are available in the wave specific technical reports [17]. Banks et al. demonstrated that respondents aged 55–64 year olds with low education background were more likely to drop out in ELSA, while there is no indication of socioeconomic status bias in attrition for respondents aged 65 years and older [18].

In this study, we had two groups of sample: treatment and control groups. For the treatment group, we included respondents from Wave 1 aged 50 years and older who had cataract surgery between Waves 2 (2004) and 6 (2012) and who responded to at least three waves of ELSA. In this way, we were able to ensure that we had information from each respondent at least one wave before and after cataract surgery. The final sample of this group consisted of 2,068 individuals. The control group consisted of respondents from Wave 1, who had no cataract disease until the latest wave they joined the survey (Wave 7), and who responded to at least three waves of ELSA. For comparability with the treatment group, we included respondents with the same age range (50 years and older) and joined the ELSA for at least three waves. This group consisted of 3,636 respondents. Ethical review for this study has been granted by the Ethical Review in H2020 panel number 668468_Sense-cog.

### Measures

Cognitive function was measured using episodic memory scores. In the memory test, the interviewers read a list of 10 simple nouns once. The respondents were asked to repeat those nouns twice: immediately after the words were read out (immediate recall) and at the end of the cognitive function module (delayed recall). The raw total scores of both tests correspond to the number of words that each respondent recalled, with a maximum score of 20. This measure is known to have good validity, and it relates to the everyday activities of older people [19].

We created a dummy variable for cataract surgery (1 for a treated respondent, 0 for a non-treated respondent). In total, 2,068 respondents received cataract surgery during the thirteen-year period based on self-reported information; we considered these respondents to be untreated before cataract surgery and treated afterwards. Our knowledge of the timing of the cataract surgery is based on self-definition by means of the question: ‘Since we last talked to you on [date], have you had cataract surgery?’. This question was posed to all respondents who considered themselves to have cataracts (ever diagnosed as having cataracts by a health professional).

The covariates included in this study were represented by demographic and socio-economic information as well as health behaviour and the presence of associated medical conditions that had been identified as risk factors for cognitive decline in a prior study [20]. Age was measured in years, while gender differentiated females from males. The socio-economic determinants included in this study were education, marital status, and wealth. We categorised education into three levels: less than high school as the reference, high school diploma, and college degree. Marital status was categorised as single, married or cohabiting as the reference, divorced, or widowed. We calculated quintiles of wealth each year to measure respondents’ ongoing economic situations, using the poorest quintile as the reference. We categorised respondents as engaging in moderate or vigorous physical activity if they did so more than once a week. Depressive symptoms were measured in all waves using the eight-item Centre for Epidemiologic Studies Depression Scale (CES-D) [21]. A set of indicators regarding comorbidities based on positive medical history (self-report of ‘has been diagnosed by a professional’) was included, covering heart attack, high blood pressure, lung disease, diabetes, stroke and cancer.

### Statistical analyses

After calculating the descriptive characteristics, we conducted propensity score matching analysis with a standard deviation of 0.1 to match the individuals on the basis of their probability of having cataract surgery conditional on all observed variables. To calculate the propensity scores, we selected seven potential variables, including gender, age, marital status, wealth, smoking behaviour, mobility and number of comorbidities confounders in a probit model that could theoretically be associated with the having the cataract surgery. In addition, including variable age to calculate the propensity score matching will control the age differences between treatment and control groups (see Table 1). S1 Table describes the covariates included in the probit model used to estimate the propensity score. The covariates are as recorded in the baseline. The findings from the probit model show that age and gender are associated with having cataract disease and cataract surgery. We used caliper matching method to match the treatment and control groups (i.e. respondents with cataract surgery and no cataract). S2 Table shows the summary statistics of the propensity score estimated by probit model in S1 Table by cataract surgery. This shows the common support region ranges from 0.06 to 0.94. The mean propensity score value for both treatment and control groups is the average probability of any individual included in the analysis, which is 0.3. The mean propensity score of treatment group (0.41) is higher than the control group (0.25) and the t-test of the difference of two group means is statistically significant (p<0.001). The distributions and region of common support of propensity scores for both the treatment (upper plot) and retired (lower plot) comparison group are shown in the S1 Fig.

**Table 1:** Baseline characteristics of treatment and control groups.

	Treatment group N = 2,068	Control group N = 3,636
Episodic memory scores	9.42 ± 3.40	10.29 ± 3.32
Age	68.54 ± 9.56	60.12 ± 8.30
Female	62.72	48.73
**Education:**		
Primary school or less	43.43	31.24
Secondary school	15.25	19.27
College or higher	41.31	49.49
**Marital status:**		
Single	5.06	5.43
Married	61.09	72.88
Divorce	9.49	12.37
Widowed	24.36	9.33
Employed	23.11	54.51
Having moderate physical activity more than once a week	58.55	63.70
Having vigorous physical activity more than once a week	14.79	23.02
Depression score	1.62 ± 1.95	1.34 ± 1.88
Number of comorbidities	0.77 ± 0.83	0.49 ± 0.69

**NOTE.** Reported are weighted mean ± SD or percentage.

Lastly, to identify differences in cognitive trajectories pre- and post-cataract surgery, we modelled cognitive trajectories by using a growth curve model, with random intercept and slope, taking episodic memory scores as the dependent variable. The growth curve model was used to adjust for clustering in the data (repeat observations within individuals) and to obtain parameter estimates alongside their standard errors. For the treatment group, we used a spline model with a knot at cataract surgery and assessed whether the pre-surgery slope differed to the post-surgery slope. We used the model with the cataract surgery variable (coded as 1 post-cataract surgery and 0 prior to cataract surgery) and its interaction with the slope term (age) to test the differences in cognitive trajectories before and after undergoing cataract surgery. We quantified the associations between cataract surgery, age, age interaction with cataract surgery and cognitive function and included demographic and socio-economic determinants (age, gender, education, marital status, and employment status), physical activity behaviour, depression, and the number of chronic diseases as the covariates. For the control group, we put an “artificial” intervention point at the point that the matched person has their surgery. We used the spline models with a knot at the “artificial” intervention knot to examine whether the pre-intervention slope differed to the post-intervention slope. We excluded the sample with no wave before or after pseudo intervention and the final sample in the control group consisted of 3,636 respondents. ELSA provided cross-sectional and longitudinal sampling weighting to adjust for non-response bias [17]. We used sampling weights for all descriptive statistics to adjust for non-response and to ensure population representativeness. We did not apply the longitudinal sampling weight for our spline analysis as it only defined for respondents who took part in all seven waves [22]. We preferred to include all respondents in wave 1 who participated also in wave 7 regardless they had missed one or more waves between waves 1 and 7. ELSA data are publicly available at http://discover.ukdataservice.ac.uk. Statistical analysis was performed using STATA Version 14 and Latent Gold Version 5.1.

## Results

A total of 2,068 individuals from the ELSA sample who underwent cataract surgery between Waves 2 and 6 and 3,636 individuals with no cataract were initially included in this study. The descriptive statistics (see Table 1) show that the respondents in the treatment group achieved an average score of nine words out of 20 in the first wave, while those in the control group were able to memorise 10 words on average. The average age of the respondents in the treatment and control groups at baseline was 68.54 and 60.12, respectively. The majority of respondents were married in both groups. More than half of the respondents in the treatment group engaged in moderate physical activity, and 14.79% of them engaged in vigorous physical activity more than once a week. The proportions of respondents in the control group who did moderate and vigorous physical activities were higher than those in the treatment groups. The average CES-D score of the respondents in the treatment and control groups at baseline were 1.62 and 1.34 on a scale of 0 to 8, with 0 representing the lowest level of depression.

Table 2 gives the parameter estimates of the three models for the slope of episodic memory scores before and after cataract surgery in the treatment and control groups. Focusing on the treatment group, cataract surgery was associated with improved memory (β = 4.23, *p*<0.001). The decline of episodic memory scores was slower after cataract surgery (β = -0.05, *p*<0.001) than before cataract surgery (β = -0.1, *p*<0.001) where social determinants, behavioural risk factors, depression score and chronic conditions were included. In the control group, the slope of cognitive decline pre-intervention in this group (β = -0.08, *p*<0.001) was gentler than that in the treatment (those with cataract surgery) group (β = -0.1, *p*<0.001). The rate of memory decline post-intervention was similar in both the control group (β = -0.05, *p*<0.001) and the treatment group (β = -0.05, *p*<0.001). These findings indicate that the cognitive trajectory of respondents with cataract disease after having cataract surgery decreased at a similar rate with those free from cataract disease.

**Table 2:** Growth curve analysis results for treatment and control group.

	Treatment group	Control group
Intercept	14.96 (0.48)**	13.84 (0.35)**
Age (before cataract surgery)	-0.1 (0.00)**	-0.08 (0.00)**
Age (after cataract surgery)	-0.05 (0.00)**	-0.05 (0.00)**
Cataract surgery	4.23 (0.41)**	4.17 (0.29)**
Female	0.69 (0.11)**	0.87 (0.07)**
**Marital status**, ref: married		
Single	-0.22 (0.22)	-0.11 (0.15)
Divorced	0.01 (0.15)	0.01 (0.09)
Widowed	-0.04 (0.09)	-0.12 (0.09)
**Education**, ref: less than high school		
High school	1.66 (0.16)**	1.68 (0.1)**
College or higher	1.77 (0.13)**	1.97 (0.08)**
**Wealth**, ref: 1^st^ quintile (poorest)		
2^nd^ quintile	0.18 (0.09)*	0.08 (0.06)
3^rd^ quintile	0.4 (0.1)**	0.39 (0.07)**
4^th^ quintile	0.55 (0.1)**	0.49 (0.07)**
5^th^ quintile (wealthiest)	0.58 (0.11)**	0.65 (0.08)**
Employed	-0.14 (0.09)	-0.06 (0.05)
Having moderate physical activity more than once a week	0.21 (0.05)**	0.27 (0.04)**
Having vigorous physical activity more than once a week	0.11 (0.08)	0.07 (0.05)
Depression score	-0.08 (0.01)**	-0.07 (0.01)**
Number of comorbidities	-0.14 (0.03)**	-0.07 (0.03)*

**NOTE.** Reported are coefficients (standard errors).

*p < .05.

**p < .001.

Several potential confounders and socio-demographic characteristics showed significant associations with episodic memory scores. Female sex, higher educational attainment, higher income and moderate physical exercise more than once a week were associated with higher memory scores. Depression and the presence of chronic diseases were negatively associated with memory scores.

**Fig 1:**
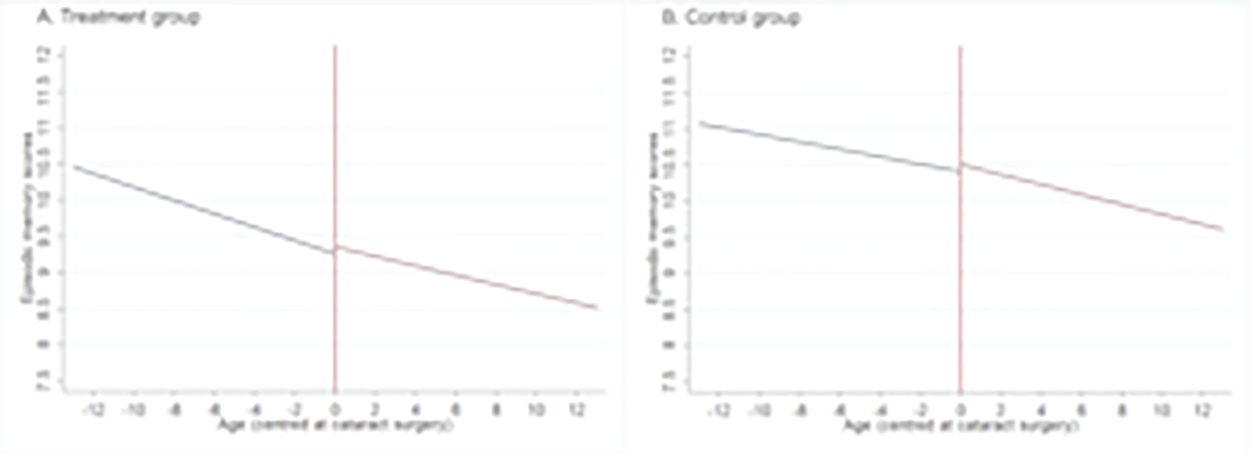
Predicted values of episodic memory before and after cataract surgery (age centred at having cataract surgery) for treatment and group controls.

Fig 1 plots the predicted trajectories of episodic memory scores before and after cataract surgery in the treatment and control groups. It is centred at the age at the time of cataract surgery for each individual. Lines before the centre of the graph show the rate of change in episodic memory score in the years leading up to the surgery, and lines after the centre of the graph show the rate of change in episodic memory score following the surgery. The model is adjusted for all covariates included in the final model. For individuals in both treatment and control groups, there were declines in episodic memory scores leading up to cataract surgery, but the decline was steeper in the treatment group. The episodic memory scores continued to decline post-surgery; however, the rate of decline was less steep in the treatment group. The rates of cognitive decline in the treatment and control groups were similar post-surgery.

## Discussion

We found cataract surgery to be associated with a reduction in the rate of cognitive decline over 13 years of follow-up. The rate of cognitive decline among individuals with cataract was gentler after than before the surgery and had become similar with that among individuals with no cataract. Our finding supports the cascade hypothesis [4, 5], according to which cataract surgery may allow better visual input and thus result in a slower rate of cognitive decline by means of several potential mechanisms. One possible mechanism is neurobiological. Better visual acuity may reduce the adverse impacts of sensory deprivation on brain function via diaschisis [23]. Neuroimaging study suggests that sensory impairment may affect brain regions distant from those in which the impairment has occurred [24]. The Baltimore Study of Aging reported that individuals with hearing impairment showed accelerated atrophy in the whole brain, particularly in the right temporal lobe [24]. Other mechanisms that may account for the positive impact of visual function on cognition include visual function’s facilitating of increased physical activity, richer social networks, better mood and higher self-efficacy. Better vision may facilitate physical activity [25], which in turn improves cognitive function [26]. Better visual function may also improve social networks and facilitate cognitive stimulation. Sensory impairment may lead to social isolation [27]; prior study has demonstrated associations between poor social networks and cognitive decline and dementia [28]. Better visual function may also lead to better mood, reducing dementia risk. Gray et al. found lower depression and anxiety levels in 92 patients at two and six months after cataract surgery [29]. Depression has been reported to be associated with cognitive impairment, including executive function and memory [30].

Dawes et al. postulated that hearing function among older adults may improve cognitive performance by increasing self-efficacy [6]. Self-efficacy is the belief in one’s own ability to accomplish tasks or succeed in specific situations. Self-efficacy impacts performance on a range of activities, including cognitive tests and measures of memory function [31]. Future studies may seek to determine whether there is a direct causal relationship between cataract surgery and cognitive performance or whether the impact of cataract surgery on cognitive performance is mediated by self-efficacy, social isolation, physical activity, depression, or a combination of those factors.

The gentler decline of cognitive trajectory after cataract surgery evidenced in our study is in keeping with the findings of previous observational and interventional studies. A previous study in England found an improvement of cognitive abilities after cataract surgery among older adults with normal cognition [14]. A study in Japan using data from 20 patients aged 61–90 with cognitive impairment found an increase in cognitive function from 12.5 points to 16.6 points out of a maximum of 30 on the revised Hasegawa dementia scale (HDS-R) after cataract surgery [32]. This finding was supported by a recent study, also from Japan [15]. There is an outside possibility that the slower rate of cognitive decline after cataract surgery in our study simply reflects the presence of a confounding effect, for example, that improved visual function after cataract surgery leads to higher cognitive test performance due to improved visibility of the cognitive test materials [7, 8]. The cognitive tests, however, were all presented verbally; the simple explanation that improved vision facilitated better performance on the cognitive tests is therefore very unlikely.

### Strengths and limitations of the study

This study has several strengths. Firstly, the repeated assessment of episodic memory over 13 years and the availability of data on cataract surgery across the same time span allowed us to examine trajectories of cognitive function before and after cataract surgery. Prior studies relied on a single cognitive assessment after surgery to evaluate the effect of cataract surgery on cognitive function [11–13], with cognitive function tested no longer than two years after surgery. One study from England twice assessed respondents’ cognitive ability post-cataract surgery [29], but the second assessment took place just six months after surgery. Age-related cognitive decline occurs gradually and accumulates during the life span [33]. Single assessments, especially those with short-term follow-ups, may not allow examination of cognitive trajectories after cataract surgery.

Secondly, ours is the first study to use a nationally representative sample to examine the association between cataract surgery, cognitive function and cognitive decline. Among the limited number of studies evaluating the effect of cataract surgery on cognitive function, only Grodstein et al. used a large sample (16,197) in 1995–2000 [11]. However, Grodstein and colleagues study included only female nurses, limiting scope for generalisation.

Finally, following the gold-standard methodology for studies of cognitive ageing [20], we used the growth curve model for modelling cognitive trajectories in order to accommodate multiple observations of an individual over time. The cognitive trajectories are influenced by natural heterogeneity among individuals due to genetic, social and behavioural factors. This methodology addresses the unobserved heterogeneity problem by allowing the discovery of individual characteristics that can explain these inter-individual differences in changes in cognitive function over time, thus enabling a robust test model for the impact of cataract surgery on the pace of cognitive decline over time.

This study has several limitations. The first limitation of this study concerns its observational design, which prevents us from interpreting the associations between cataract surgery and cognitive ability as causal. Despite the wide range of potential confounders included in our analysis, other unmeasured variables could prove important. Future randomised controlled trials are required to confirm causality. Second limitation is that episodic memory as the main outcome does not definitely measure cognitive ageing, given that it is only one among many cognitive functions that change with age [34]. Furthermore, the age-related changes in those different cognitive abilities occur at different rates [35]. Declining episodic memory, however, is one of the earliest indicators of neurodegenerative disorders [36], and episodic memory is important for financial decision-making in later life [37]. Thirdly, we have no information as to whether the respondents underwent cataract surgery on one eye only or on the second eye. The differences in vision between the operated and unoperated eyes in the respondents who had cataract surgery on only one eye may have affected their cognitive ability. However, a recent study in England reported that the improved visual function of patients who underwent second-eye cataract surgery was comparable to that of first-eye patients. It further found that cataract surgery in both first- and second-eye patients was significantly associated with better cognitive function six months after the surgery [29]. Finally, the information regarding the presence of cataract disease and cataract surgery used in this study was based on self-report only, which may have been influenced by recall bias. Cataract disease is common, and respondents in the control group may also have a cataract, which either was not diagnosed, or they did not recall their diagnosis. Moreover, while people are unlikely to say that they had cataract surgery, some may not recall that they had cataract surgery. This limitation may have reduced differences between the treatment and control groups. Future data collection is needed to improve information of individual past experiences.

## Conclusions

Cataract surgery was positively associated with a lower rate of cognitive decline among older adults in England, independent of risk factors for cognitive impairment including those related to age, gender, education, wealth, chronic diseases, depressive symptoms, and physical inactivity. Due to the high prevalence of both cataracts and cognitive disorders in later life, the association between cataract surgery and cognitive longevity has important public health implication for targeting people at risk of cognitive decline and as a potential means to improve cognitive health and prevent dementia. A recent study using ELSA showed that the odds ratio for future pathological cognitive impairment was more than five times lower among respondents with the most advantaged trajectory of episodic memory than among those in the most disadvantaged trajectory [38]. A positive impact of cataract surgery on cognitive decline would support the presence of a direct or indirect causal impact of visual impairment on cognitive ageing. Further research may test the potential for treatment and/or prevention of vision impairment to lower the risk of dementia.

## Supporting information

S1 Fig Distributions and region of common support of propensity scores for individuals who had cataract surgery (upper plot) and with no cataract disease (lower plot) comparison group
(TIF)

S1 Table Results of the probit model to estimate the propensity score matching for cataract surgery. (DOCX)

S2 Table Summary statistics of the propensity scores. (DOCX)

S3 Table Mean of episodic memory in each wave before and after intervention/pseudointervention. (DOCX)
