# Correction: Cataract surgery and age-related cognitive decline: A 13-year follow-up of the English Longitudinal Study of Ageing

**DOI:** 10.1371/journal.pone.0208045

**Published:** 2018-11-20

**Authors:** Asri Maharani, Piers Dawes, James Nazroo, Gindo Tampubolon, Neil Pendleton, on behalf of the SENSE-Cog WP1 group

The following information is missing from the Funding section: PD is supported by the NIHR Manchester Biomedical Research Centre.

## References

[1] Maharani A, Dawes P, Nazroo J, Tampubolon G, Pendleton N, on behalf of the SENSE-Cog WP1 group (2018) Cataract surgery and age-related cognitive decline: A 13-year follow-up of the English Longitudinal Study of Ageing. PLoS ONE 13(10): e0204833 10.1371/journal.pone.0204833 30307960 PMC6181298

