# Supplementary figures and images for: Cataract surgery and age-related cognitive decline: A 13-year follow-up of the English Longitudinal Study of Ageing
Source: PLoS One. 2018 Oct 11;13(10):e0204833. doi: 10.1371/journal.pone.0204833 (PMC6181298; doi:10.1371/journal.pone.0204833)

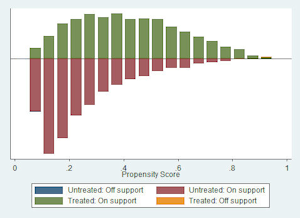

Supplement: S1 Fig — Distributions and region of common support of propensity scores for individuals who had cataract surgery (upper plot) and with no cataract disease (lower plot) comparison group (TIF) [file pone.0204833.s001.tif]
