# Supplementary material for: Cataract surgery and age-related cognitive decline: A 13-year follow-up of the English Longitudinal Study of Ageing
Source: PLoS One. 2018 Oct 11;13(10):e0204833. doi: 10.1371/journal.pone.0204833 (PMC6181298; doi:10.1371/journal.pone.0204833)
Supplement: S1 Table — (DOCX) [file pone.0204833.s002.docx]

**S1 Table** Results of the probit model to estimate the propensity score matching for cataract surgery

| **Covariate** | **Coeff (SE)** | **p-value** |
| --- | --- | --- |
| Intercept | -4.09 (0.14) | <0.001 |
| Female | 0.32 (0.03) | <0.001 |
| Age | 0.04 (0.00) | <0.001 |
| Married | -0.07 (0.03) | 0.061 |
| Wealth (in quintile) | 0.03 (0.01) | 0.006 |
| Past smoker | 0.07 (0.03) | 0.046 |
| Current smoker | -0.08 (0.05) | 0.092 |
| Mobility | 0.03 (0.00) | <0.001 |
| Number of comorbidities | 0.1 (0.02) | <0.001 |
