# Supplementary material for: Cataract surgery and age-related cognitive decline: A 13-year follow-up of the English Longitudinal Study of Ageing
Source: PLoS One. 2018 Oct 11;13(10):e0204833. doi: 10.1371/journal.pone.0204833 (PMC6181298; doi:10.1371/journal.pone.0204833)
Supplement: S2 Table — (DOCX) [file pone.0204833.s003.docx]

**S2 Table** Summary statistics of the propensity scores

|  | **Frequency** | **Mean (SD)** | **Min.** | **Max.** |
| --- | --- | --- | --- | --- |
| All | 6675 | 0.30 (0.18) | 0.06 | 0.94 |
| No cataract | 4631 | 0.25 (0.16) | 0.06 | 0.94 |
| Cataract surgery | 2044 | 0.41 (0.19) | 0.07 | 0.94 |
