# Supplementary material for: Cataract surgery and age-related cognitive decline: A 13-year follow-up of the English Longitudinal Study of Ageing
Source: PLoS One. 2018 Oct 11;13(10):e0204833. doi: 10.1371/journal.pone.0204833 (PMC6181298; doi:10.1371/journal.pone.0204833)
Supplement: S3 Table — (DOCX) [file pone.0204833.s004.docx]

**S3 Table** Mean of episodic memory in each wave before and after intervention/pseudointervention

| **Wave** | **Treatment** | **Control** |
| --- | --- | --- |
| 5 waves before cataract surgery | 10.2 | 10.7 |
| 4 waves before cataract surgery | 10.3 | 11 |
| 3 waves before cataract surgery | 10.1 | 10.9 |
| 2 waves before cataract surgery | 9.8 | 10.7 |
| 1 waves before cataract surgery | 9.5 | 10.5 |
| Waves at cataract surgery | 9.5 | 10.4 |
| 1 waves after cataract surgery | 9.5 | 10.3 |
| 2 waves after cataract surgery | 9.1 | 10.3 |
| 3 waves after cataract surgery | 9 | 10.2 |
| 4 waves after cataract surgery | 8.8 | 10 |
| 5 waves after cataract surgery | 8.9 | 9.8 |
